# Hospital Epidemics Tracker (HEpiTracker): Description and pilot study of a mobile app to track COVID-19 in hospital workers

**DOI:** 10.2196/21653

**Published:** 2020-09-21

**Authors:** Joan B Soriano, Esteve Fernández, Álvaro de Astorza, Luis A Pérez de Llano, Alberto Fernández-Villar, Dolors Carnicer-Pont, Bernardino Alcázar-Navarrete, Arturo García, Aurelio Morales, María Lobo, Marcos Maroto, Eloy Ferreras, Cecilia Soriano, Carlos Del Rio-Bermudez, Lorena Vega-Piris, Xavier Basagaña, Josep Muncunill, Borja G Cosio, Sara Lumbreras, Carlos Catalina, José María Alzaga, David Gómez Quilón, Carlos Alberto Valdivia, Celia de Lara, Julio Ancochea

**Affiliations:** 1 Hospital Universitario La Princesa Universidad Autónoma de Madrid Madrid Spain; 2 Centro de Investigación en Red de Enfermedades Respiratorias (CIBERES) Instituto de Salud Carlos III (ISCIII) Madrid Spain; 3 Unidad de Control del Tabaco Centro Colaborador de la OMS para el Control del Tabaco, Institut Català d'Oncologia-ICO Hospitalet de Llobregat Barcelona Spain; 4 Grupo de Control y Prevención del Cáncer Institut d'Investigació Biomèdica de Bellvitge-IDIBELL Hospitalet de Llobregat Barcelona Spain; 5 Departamento de Ciencias Clínicas Facultad de Medicina, Universitat de Barcelona Hospitalet de Llobregat Barcelona Spain; 6 Servicio de Neumología Hospital de Can Misses Ibiza Spain; 7 Servicio de Neumología Hospital Lucus Augusti Lugo Spain; 8 Servicio de Neumología Hospital Álvaro Cunqueiro Vigo Spain; 9 Servicio de Neumología Hospital de Alta Resolución de Loja Loja Spain; 10 Fundación Teófilo Hernando Madrid Spain; 11 CIMNE - International Center for Numerical Methods in Engineering Barcelona Spain; 12 SAVANA Med Madrid Spain; 13 Unidad de Metodología IIS del Hospital Universitario La Princesa Madrid Spain; 14 ISGlobal Universitat Pompeu Fabra Barcelona Spain; 15 IdISBa Palma Spain; 16 Servicio de Neumología Hospital Universitari Son Espases Palma Spain; 17 Institute for Research in Technology Universidad Pontificia Comillas Madrid Spain; 18 ASELCIS Consulting Madrid Spain

**Keywords:** app, COVID-19, coronavirus, e-medicine, monitoring, symptoms, surveillance

## Abstract

**Background:**

Hospital workers have been the most frequently and severely affected professional group during the COVID-19 pandemic, and have a big impact on transmission. In this context, innovative tools are required to measure the symptoms compatible with COVID-19, the spread of infection, and testing capabilities within hospitals in real time.

**Objective:**

We aimed to develop and test an effective and user-friendly tool to identify and track symptoms compatible with COVID-19 in hospital workers.

**Methods:**

We developed and pilot tested Hospital Epidemics Tracker (HEpiTracker), a newly designed app to track the spread of COVID-19 among hospital workers. Hospital staff in 9 hospital centers across 5 Spanish regions (Andalusia, Balearics, Catalonia, Galicia, and Madrid) were invited to download the app on their phones and to register their daily body temperature, COVID-19–compatible symptoms, and general health score, as well as any polymerase chain reaction and serological test results.

**Results:**

A total of 477 hospital staff participated in the study between April 8 and June 2, 2020. Of note, both health-related (n=329) and non–health-related (n=148) professionals participated in the study; over two-thirds of participants (68.8%) were health workers (43.4% physicians and 25.4% nurses), while the proportion of non–health-related workers by center ranged from 40% to 85%. Most participants were female (n=323, 67.5%), with a mean age of 45.4 years (SD 10.6). Regarding smoking habits, 13.0% and 34.2% of participants were current or former smokers, respectively. The daily reporting of symptoms was highly variable across participating hospitals; although we observed a decline in adherence after an initial participation peak in some hospitals, other sites were characterized by low participation rates throughout the study period.

**Conclusions:**

HEpiTracker is an already available tool to monitor COVID-19 and other infectious diseases in hospital workers. This tool has already been tested in real conditions. HEpiTracker is available in Spanish, Portuguese, and English. It has the potential to become a customized asset to be used in future COVID-19 pandemic waves and other environments.

**Trial Registration:**

ClinicalTrials.gov NCT04326400; https://clinicaltrials.gov/ct2/show/NCT04326400

## Introduction

The rapid spread of severe acute respiratory syndrome coronavirus 2 (SARS-CoV-2), the virus responsible for COVID-19, requires an urgent, collaborative, and multidisciplinary response supported by innovative methods [1]. Hospital staff (including both health-related and non–health-related professionals) form the backbone of the response to the ongoing pandemic. However, these professionals are among the most frequently and severely affected by COVID-19 [2,3]. Indeed, the disease has had a tremendous impact on the hospital workforce of affected areas due to the high risk of infection and heavy workloads. The dissemination of SARS-CoV-2 within hospitals may result in large nosocomial outbreaks and other devastating consequences. In the current scenario, timely information on how these risks evolve and are managed is almost anecdotal and reliable scientific data are urgently needed. In addition, understanding the determinants of SARS-CoV-2 infection and transmission by individuals with asymptomatic or very mild symptomatic cases of COVID-19 is crucial for the design of containment strategies.

In August 2020, the World Health Organization (WHO) declared that the COVID-19 pandemic is far from controlled. The cumulative number of confirmed COVID-19 cases across 216 countries, areas, or territories worldwide amounts to over 21,989,366, and 775,893 confirmed deaths have been reported to date [4]. Record daily numbers of both infections and deaths are seen in many countries, with many of them already experiencing “second waves” after lockdowns were lifted [5]. Spain is among the countries hardest hit by the pandemic, with over 376,000 total cases and over 28,000 deaths as of August 2020 [6].

COVID-19–related symptoms are nonspecific, resembling common cold symptoms in immunocompetent individuals. According to the Centers for Disease Control and Prevention (CDC) [7], the list of common COVID-19 symptoms includes fever, cough, and shortness of breath that may appear 2 to 14 days after exposure to SARS-CoV-2; other nonrespiratory symptoms are also frequent [8]. Whenever these symptoms appear with epidemiological evidence (ie, after close contact with an infected subject or after visiting an area with ongoing community spread), further clinical assessment is needed.

The real-time assessment of COVID-19–related symptoms, their spread, and testing capabilities in hospital settings requires the use of innovative tools. In this context, digital health technologies have great potential to improve surveillance and epidemic control, primarily through increased information coverage, faster acquisition and distribution of information, rapid case tracking, and improved proximity tracing [9-11]. Consequently, smartphone- and web-based health apps aimed at tracking COVID-19 are on the rise. Although digital tools can promote public health, they can be intrusive, erode individual freedoms, or leave vulnerable populations behind [12].

Here, we summarize the development of Hospital Epidemics Tracker (HEpiTracker) [13], a newly designed app to track COVID-19 and other epidemics in hospitals. We also describe the pilot study performed across different areas and phases of the outbreak. The goal of the app is to help already overwhelmed hospital staff to actively monitor and assess COVID-19 infections and compatible symptoms in a population of hospital workers. We provide the basic data of the app and descriptive statistics of the pilot study, which illustrate the applicability of HEpiTracker in practical settings.

## Methods

### Overview

On March 14, 2020, a multidisciplinary group of individuals with varied backgrounds held the first of many daily meetings to discuss, by means of a “think tank” approach, research avenues aimed at mitigating the impact of the COVID-19 crisis. As part of the Active Monitoring And Determinants of Incident Infection of COVID-19 in a Hospital population (AMADIICH) initiative, this multidisciplinary group created a framework designed to collect large amounts of heterogeneous data regarding COVID-19 in hospital staff, from shoe-leather epidemiology to big data and biosensors [14-16]. One of the main priorities of the group was speed, and the tools were designed with the aim of being applied during the first wave of COVID-19, and any subsequent outbreaks. Ethics approval of the AMADIICH research protocol was granted by the University Hospital of la Princesa’s ethics board on March 19, 2020 (Proceedings of the Standing Commission CEIm 02/20, registry number 4061). The study was registered on ClinicalTrials.gov with the identiﬁer NCT04326400. Individual informed consent was a requirement of participation and was obtained on the first screen of the HEpiTracker app, with tick boxes to give or deny consent.

The standard process of reporting COVID-19–related symptoms differs by area and center, but typically starts with a phone call from the employee to the occupational health unit (OHU) of the center. The employee is then advised to self-isolate at home, where he/she receives a polymerase chain reaction (PCR) test. If the test is positive, he/she remains in home isolation. If the test is negative but there are symptoms, the employee remains in isolation and the test is repeated during the following days. Therefore, workers can only return to work once they do not have symptoms and have returned two consecutive negative PCR tests. The OHU should always have access to the status of all employees and is typically responsible for escalating the data. The purpose of the HEpiTracker app is to provide an easier, homogenous, and transparent way of tracking positive PCR results and symptoms. The tracking happens automatically and relieves the health manager from manually updating the aggregated information and calculating statistics. It can also provide the updated information to the workers themselves in a transparent manner.

We provide descriptive data obtained in the pilot study that illustrate the applicability of the app in practical settings. We would like to clarify that we do not intend to study the factors behind app adoption or the effect these types of tools have on infection rates. These, and other related issues, are beyond the scope of this article. What we do present is a working app that can help already overwhelmed hospital staff to actively monitor and assess COVID-19 infections and compatible symptoms in the hospital worker population.

### App Development Process

As mentioned above, a mobile app to help monitor the spread of COVID-19 within hospitals was conceived after a state of emergency and full lockdown were declared in Spain on March 14, 2020. Following initial discussions and ethical approval, a stepwise approach was carried out by ASELCIS software developers [17] to create the first version of the new app within a week and then to enhance its functionalities regularly. After several iterations, a minimum set of variables to include in the HEpiTracker App were identified, including demographic and occupational data, symptoms, previous comorbidities, and lab testing variables (Table 1).

There was a feedback process from users within our scientific committee, which includes doctors, nurses, computer science specialists, mathematicians, physicists, and statisticians, but not patients themselves, although during the development of the app several authors became infected or were quarantined due to COVID-19. The app also included a self-assessment of overall health status based on an ordinal Likert scale from 0 to 10. HEpiTracker was made available for both Android and iOS operating systems at Google and Apple stores, respectively.

Once HEpiTracker was up and running, we designed a pilot study in real-world conditions to test the feasibility of the app. Specifically, we tested the app in several hospitals across regions with different incidence rates and undergoing different phases of the COVID-19 pandemic: Hospital Can Misses (Eivissa) from April 9, 2020; Hospital Lucus Augusti (Lugo) from April 10, 2020; Hospital Álvaro Cunqueiro (Vigo) from April 10, 2020; Hospital Institut Català d'Oncologia (ICO; l’Hospitalet, Badalona, Girona, Tarragona-Terres de l’Ebre) from April 8, 2020; Hospital de Alta Resolución Loja (Granada) from April 13, 2020; and Hospital Universitario de La Princesa (Madrid) from April 9, 2020.

Hospital staff in 5 Spanish autonomous communities (Andalusia, Balearics, Catalonia, Galicia, and Madrid) were invited to download the app on their smartphones [13], and to register their daily body temperature, COVID-19–compatible symptoms, and general health score, as well as any PCR or serological test results. All staff in the participating hospitals, namely doctors, nurses, technicians, administrative workers, wardens, cleaners, managers, cafeteria staff, security, and other occupations were invited to participate, with no exclusion criteria.

**Figure 1:**
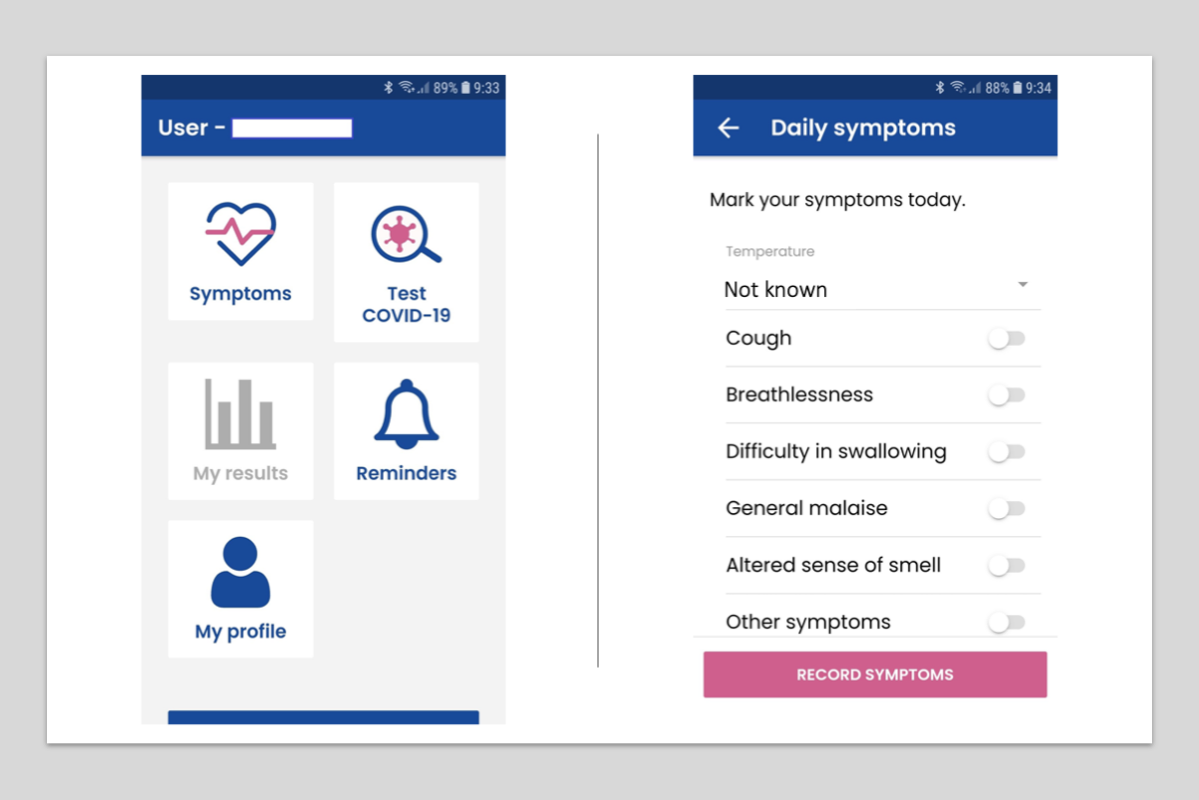
HEpiTracker graphical display and screens.

In addition to answering Yes/No for the presence of daily symptoms, participants self-assessed their overall health by means of a visual analog scale (VAS), and they disclosed whether they had a history (either of diagnosis or treatment) of rhinitis, allergy, or chronic obstructive pulmonary disease (COPD)/chronic bronchitis, as well as their smoking status. Further, participants manually entered their body temperature in degrees Celsius to one decimal. They were also invited to register the outcome and the date of any COVID-19 laboratory test (PCR, IgG, or IgM); these could have been performed routinely at their center, throughout the study by risk exposure, or as a result of the presence of symptoms or suspicion of having the disease (Figure 1).

**Table 1:** Variables included in the HEpiTracker App.

Type and variable		Values
**Demographic data**		
	Personal ID	DNI/NIE^a^ and email
	Age (years)	18-122
	Sex	Male/Female
**Occupational data**		
	Current job category	Physician, nurse, technician, administrative, warden, cleaner, manager, cafeteria, security, other^b^
	Department	Service^c^
**Symptoms**		
	Body temperature	Degrees Celsius, reported to one decimal
	Cough	Yes/No
	Shortness of breath	Yes/No
	Odynophagia or pain when swallowing	Yes/No
	Malaise	Yes/No
	Alterations of sense of smell	Yes/No
	“My health today is…”	Visual analog scale from 0 to 10
**Previous comorbidities**		
	Rhinitis	Yes/No
	Asthma	Yes/No
	Chronic bronchitis or chronic obstructive pulmonary disease	Yes/No
	Smoker	Never/former/current
**Lab testing**		
	COVID-19 test^d^	Positive or negative

^a^DNI: Documento nacional de identidad; NIE: Número de identificación de extranjero.

^b^“Other” category without text/alphanumericals.

^c^Departments/services include the following: Pathology, Cardiology, General and Digestive System Surgery, Oral and Maxillofacial Surgery, Plastic and Reconstructive Surgery, Medical-Surgical Dermatology and Venereology, Gastroenterology - Digestive System, Gynecology and Obstetrics, General Medicine, Nuclear Medicine, Preventive Medicine, Neurophysiology, Neurology, Ophthalmology, Medical Oncology, Radiation Oncology, Otorhinolaryngology, Pediatrics and Specific Areas Children's Health, Radiodiagnosis - Diagnostic Imaging, Traumatology and Orthopedic Surgery, Urology, Emergencies, restricted-COVID-19 area, quarantine area, isolation area.

^d^Information recorded includes the date and type of test: polymerase chain reaction, IgG, or IgM.

### Participant Withdrawal Criteria

A participant could withdraw from the study at any time (by simply not filling in their own information or by removing the app from their smartphone). However, given the ongoing public health emergency during this COVID-19 outbreak, it was agreed that any data already obtained would be kept for analysis and grouped tabulation. In addition, participants would be withdrawn at the discretion of the investigators if they failed to comply with the protocol procedures (eg, dummy data, relative of hospital staff, and other).

### Data Life Cycle

The first data entry was made by a user of the mobile app. The coding language is based on Ionic, which allows developers to create native apps with web coding such as HTML, CSS, and JavaScript. Users’ initial data and subsequent symptom records are automatically transferred to an Odoo V11 Enterprise Edition server application [18]. The coding language is Python 3 on the back-end and JavaScript on the front-end. This first data transfer is carried out in encrypted form with an SSL certificate and a HTTPS protocol. In this server application, the data is processed and sent to the PostgreSQL database via an SSL certificate.

### Final Data Storage

The storage of data is done in a PostgreSQL database in an encrypted way. In addition, user data is stored anonymously with an internal code assigned to each participant. In this way, the user's identification number is related to the internal code, and all data entered is linked to it, preventing the end user (principal investigator) from having access to the user's personal data.

### Backups

To guarantee the storage of data and avoid its loss or modification, a backup is made daily that is kept in three data centers (DCs) on three different continents, thus ensuring the integrity of the data in the event of any serious problem or inconvenience in any of the three DCs.

All individual participants’ collected data were stored on secure ASELCIS servers. Data were anonymized with a unique identifier by user and hospital. Statistics were performed by ISGlobal and IdIsBa with databases already anonymized over a PostgreSQL connection under a user and password requirement.

### Statistical Analysis

Study reports were sent to the participating hospitals. These reports included descriptive information regarding changes in the symptoms and incidence of COVID-19 infection by age group, sex, job category, and department/section. We followed the STROBE (Strengthening the Reporting of Observational Studies in Epidemiology) guidelines for reporting observational studies [19]. Continuous variables were expressed as mean and standard deviation, and categorical variables were expressed as number and percentage.

## Results

A total of 477 hospital staff participated in the study between April 8 and June 2, 2020 (Table 2).

Of note, both health-related (n=329) and non–health-related (n=148) professionals participated in the study. Overall participation by center was low; the highest participation rate of potential participants was 5.06% at Hospital Álvaro Cunqueiro (128 of 2529 potential participants), followed by a participation rate of 2.97% at ICO L'Hospitalet (20 of 674 potential participants). Most participants were female (67.7%), with a mean age of 45.4 years (SD 10.6). Regarding smoking habits, 13.0% and 34.2% of participants were current or former smokers, respectively (Table 2). Over two-thirds of participants (68.8%) were health workers (43.4% physicians and 25.4% nurses); however, the proportion of non–health workers by center ranged from 40% to 85%, and the distribution of job category by center was also highly variable. Participation was therefore lower for non–health workers, although we did obtain valuable data about them. Regarding comorbidities, participants reported being previously diagnosed with or currently in treatment for the following respiratory conditions: allergic rhinitis (25.4%), asthma (13.8%), and chronic bronchitis/COPD (1.0%).

**Figure 2:**
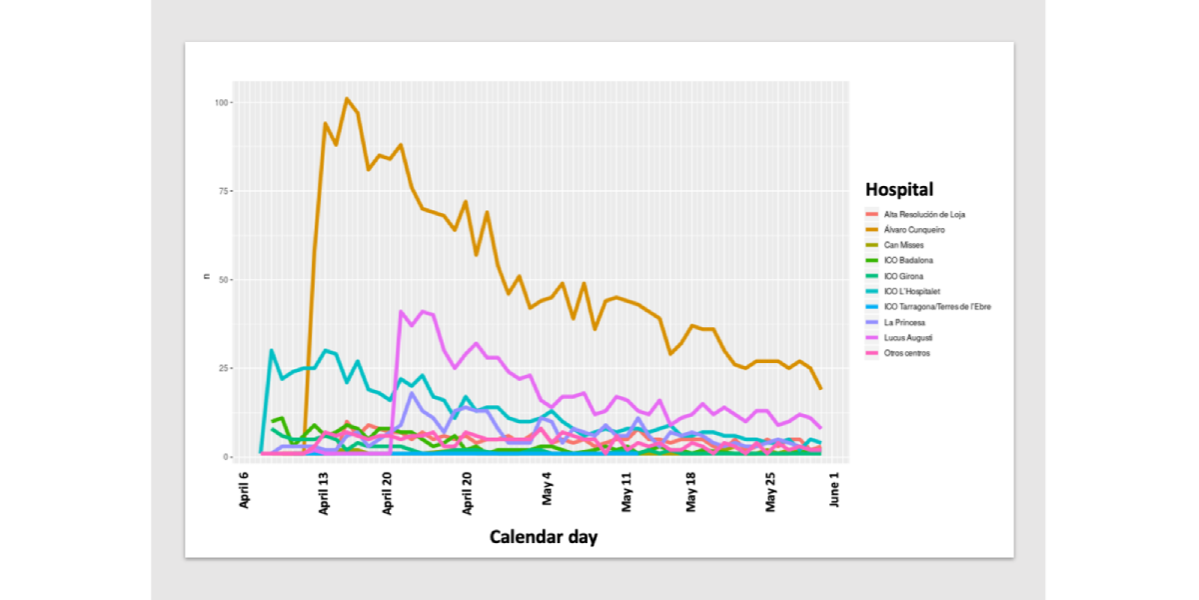
Distribution of HEpiTracker coverage in each hospital by calendar day (April 8 to May 30, 2020) as of June 2, 2020. ICO: Institut Català d'Oncologia.

The daily report of symptoms was highly variable across participants; overall, 2% to 6% of the source population in each hospital engaged with the app. Although we observed a decline in adherence after an initial participation peak in some hospitals, other sites were characterized by poor participation rates since inception and throughout the study period (Figure 2).

There were no major differences across hospitals in the distribution of respiratory comorbidities (asthma, rhinitis, and chronic bronchitis/COPD), smoking status, or symptoms, namely cough, shortness of breath, malaise, or anosmia (Table 3).

However, for temperature and overall health status scored from 0 to 10, there were subtle but not clinically significant differences. Finally, the percentage of positive PCR tests was highly variable, from 39% of participants at La Princesa (16/41 participants), to 20% at ICO Girona (2/10), 9.5% (16/169) at Álvaro Cunqueiro, 8.8% (6/68) at ICO L’Hospitalet, and 3.6% (4/116) at Lucus Augusti.

**Figure 3:**
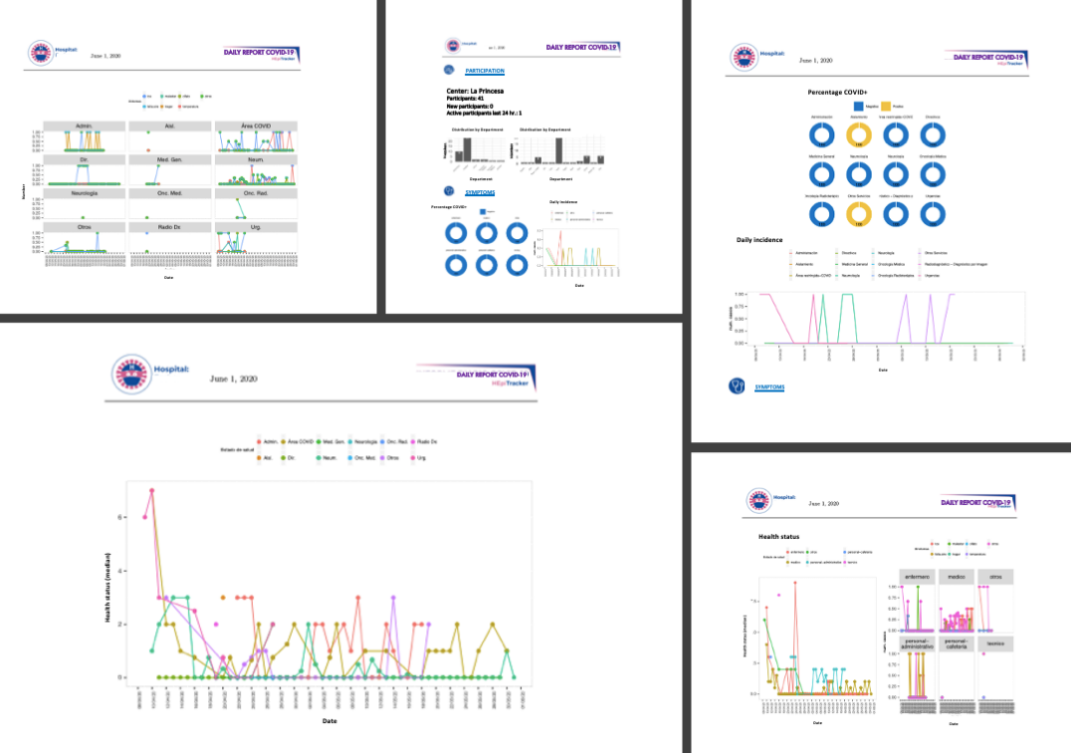
HEpiTracker results by hospital and by calendar day.

A daily summary display of these results was made available for circulation at all participating sites each morning during the study period (Figure 3).

**Table 2:** Demographic and clinical characteristics of 477 HEpiTracker users.

Characteristics			Value	
Female, n (%)			323 (67.7)	
Age (years), mean (SD)			45.4 (10.6)	
**Hospital, n (%)**				
	Hospital Can Misses (Eivissa)			11 (2.3)
	Hospital Lucus Augusti (Lugo)			112 (23.5)
	Hospital Álvaro Cunqueiro (Vigo)			169 (35.4)
	Hospital Institut Català d'Oncologia			100 (21.0)
		l’Hospitalet		68 (14.3)
		Badalona		20 (4.2)
		Girona		10 (2.1)
		Tarragona-Terres de l’Ebre		2 (0.4)
	Hospital de Alta Resolución de Loja (Granada)			20 (4.2)
	Hospital Universitario de La Princesa (Madrid)			48 (8.6)
	Other			24 (5.0)
**Job description, n (%)**				
	Physician			207 (43.4)
	Nurse			121 (25.4)
	Technician			40 (8.4)
	Administrative			38 (8.0)
	Warden			12 (2.5)
	Cleaner			7 (1.5)
	Manager			6 (1.2)
	Cafeteria			3 (0.8)
	Security			1 (0.2)
	Other			41 (8.6)
**Respiratory conditions, n (%)**				
	Allergic rhinitis			121 (25.4)
	Asthma			66 (13.8)
	Chronic bronchitis or chronic obstructive pulmonary disease			5 (1.0)
**Smoking status, n (%)**				
	Never smoker			252 (52.8)
	Former smoker			163 (34.2)
	Current smoker			62 (13.0)

**Table 3:** Distribution of HEpiTracker variables by hospital.

Variables		Alta Resol de Loja (N=20)	Álvaro Cunqueiro (N=169)	Can Misses (N=11)	ICO^a^ Badalona (N=20)	ICO Girona (N=10)	ICO L'Hospitalet (N=68)	ICO Tarragona (N=2)	La Princesa (N=41)	Lucus Augusti (N=112)	Other centers (N=24)
**Comorbidities, n (%)**											
	Asthma	3 (15.0)	29 (17.2)	3 (27.3)	2 (10.0)	2 (20.0)	7 (10.3)	0 (0.00)	4 (9.76)	10 (8.93)	6 (25.0)
	Rhinitis	4 (20.0)	52 (30.8)	6 (54.5)	3 (15.0)	1 (10.0)	12 (17.6)	1 (50.0)	11 (26.8)	23 (20.5)	8 (33.3)
	Chronic bronchitis or COPD^b^	0 (0.00)	3 (1.78)	0 (0.00)	0 (0.00)	1 (10.0)	0 (0.00)	0 (0.00)	0 (0.00)	1 (0.89)	0 (0.00)
**Smoking, n (%)**											
	Former	6 (30.0)	61 (36.1)	2 (18.2)	7 (35.0)	4 (40.0)	25 (36.8)	0 (0.0)	11 (26.8)	38 (33.9)	9 (37.5)
	Never	7 (35.0)	87 (51.5)	9 (81.8)	12 (60.0)	6 (60.0)	36 (52.9)	2 (100)	28 (68.3)	56 (50.0)	9 (37.)
	Current	7 (35.0)	21 (12.4)	0 (0.00)	1 (5.00)	0 (0.0)	7 (10.3)	0 (0.00)	2 (4.88)	18 (16.1)	6 (25.0)
**Symptoms, n (%)**											
	Cough	0 (0.00)	14 (8.28)	0 (0.00)	2 (10.0)	1 (10.0)	6 (8.82)	0 (0.00)	4 (9.76)	3 (2.68)	4 (16.7)
	Shortness of breath	0 (0.00)	2 (1.18)	0 (0.00)	1 (5.00)	0 (0.0)	3 (4.41)	0 (0.00)	2 (4.88)	0 (0.00)	2 (8.33)
	Malaise	0 (0.00)	2 (1.18)	1 (9.09)	0 (0.00)	0 (0.0)	3 (4.41)	0 (0.00)	3 (7.32)	0 (0.00)	1 (4.17)
	Anosmia	0 (0.00)	2 (1.18)	0 (0.00)	0 (0.00)	0 (0.0)	5 (7.35)	0 (0.00)	2 (4.88)	1 (0.89)	1 (4.17)
Temperature, mean (SD)		35.7 (0.58)	35.8 (0.58)	29.4 (14.5)	35.7 (0.67)	35.6 (0.53)	35.6 (0.67)	35.0 (0.00)	35.9 (0.78)	35.7 (0.65)	34.3 (7.33)
Overall health, mean (SD)		0.80 (2.28)	1.11 (2.31)	0.64 (1.80)	1.05 (2.31)	0.40 (0.84)	0.99 (1.59)	0.00 (0.00)	1.17 (2.23)	0.62 (1.47)	2.71 (3.01)

^a^ICO: Institut Català d'Oncologia.

^b^COPD: chronic obstructive pulmonary disease.

## Discussion

### Summary of Results

HEpiTracker is a newly designed mobile app aimed at monitoring the spread of COVID-19 symptoms and testing among professionals in hospital settings. Although the first wave of the pandemic in Spain and other countries is thought to be over, many experts warn that lockdown lifts might be premature [20]. In the current situation, the use of novel tools to measure and track the effects of the pandemic in real time may help tackle the forthcoming waves of the pandemic [4,6].

We tested the HEpiTracker app in a sample of 477 hospital staff including both health-related and non–health-related professionals from 9 centers in 5 regions of Spain experiencing different stages of the COVID-19 pandemic. The daily report of COVID-19–related symptoms was highly variable across participating hospitals, as well as the reported infection testing rates. We observed a decline in adherence after an initial participation peak in some hospitals, while other sites were characterized by low participation rates throughout the study period. It is worth noting that our pilot study aimed to test the technical aspects of the app in different real-world hospital settings, all in different stages of the COVID-19 pandemic, but not its deployment or coverage. In general, an acceptable response rate for any epidemiological study is 80% or higher for usability [21,22]. Having said that, the total workforce in our 9 participating hospitals ranges from around 150 to over 3000 workers, which fluctuate seasonally and yearly. As reported, the overall response rate varied from 2% to 6% of the source population in each hospital in this study.

In future analyses, techniques and tools used in artificial intelligence and machine learning will be explored. For instance, machine learning can be used to forecast new cases or to identify relevant phenotypes [20].

### Discussion of Results and Work in the Field

Mobile apps are effective, valid tools for monitoring very diverse patterns in real-life conditions [23]. However, a key issue in mobile app–based monitoring involves increasing adherence and reinforcement for changing established behaviors. Our participation data show that adherence to the app should be improved, perhaps by providing some real-time feedback, composed of aggregated data from a given user’s hospital and overall estimates, to the users. In response to the ongoing COVID-19 pandemic, several apps and digital health solutions have already been developed [24-26], as digital technology has the potential to improve surveillance and epidemic control. This is achieved primarily through increased information coverage, faster acquisition and distribution of information, rapid case tracking, and improved proximity tracing. In this context, some have already identified new opportunities to reshape current health care systems, including the widespread adoption of electronic health records and the development of better mobile health apps and other disruptive technologies [10]. Indeed, digital health solutions are a promising asset to improve the quality of health care at a more sustainable cost. In a recent review, the uptake of and engagement with health and well-being smartphone apps was associated with capability, opportunity, and motivation [27].

It should be stressed that the present study did not intend to study the factors that determine app adoption or the impact of the app on infection rates. These, along with other relevant issues, are outside the scope of this paper and would only be addressed by a larger-scale study that would be complex in its design and execution. However, this pilot study allowed us to identify some strengths and limitations of the app that will be addressed in the following sections.

### Strengths and Potential of the Platform

Some strengths of HEpiTracker include novelty, flexibility, and the ability to quickly modify it and include new updates and information. Notably, the app is now available in several languages (Spanish, English, and Portuguese) and is accepted by both health care professionals and non–health care professionals in hospital settings. In the near future, we plan to design customized versions to be used in primary care, by security forces, and even in universities once in-class teaching is resumed.

### Limitations

However, our results must be interpreted in light of the following limitations. Despite fulfilling all European Union regulations and disclaimers on data protection, concerns with data privacy were raised by legal departments or individual managers in several nonparticipating hospitals, so clarity among leadership should be ensured. When evaluating usability and user experience of mobile health (mHealth) solutions, there are standardized questionnaires such as The Standardized User Experience Percentile Rank Questionnaire (SUPR-Qm) [28] for user experience and the mHealth App Usability Questionnaire (MAUQ) [29], which can aid the evaluation of apps; these can be used to prospectively assess HEpiTracker. However, the main limitation of the study was adherence to the app. In particular, we found it difficult to maintain participant engagement for weeks, especially when the local COVID-19 situation deescalated by the end of April/May 2020.

Unfortunately, the inclusion of alarm reminders for the daily recording of symptoms and temperature was not effective. Indeed, a proper communication and marketing strategy for wider implementation will be critical for its future use. We have already developed QR codes and templates of posters to pin in hospital entrances, elevators, and notice boards, which serve as a way to download HEpiTracker directly on any platform.

This lack of adherence, however inspired the next evolution of the app, consisting of an activity wristband that will incorporate HEpiTracker plus a number of other utilities. This evolution of HEpiTracker, named Epiwrist (an “epidemiologist on your wrist”), could passively monitor all HEpiTracker variables, as well as others. Epiwrist would include a gyroscope to assess hand-washing behavior (duration and frequency), synchronized with a cough sensor to identify if the cough is directed to your sleeve (good) or your hand (bad), and a continuous heart rate monitor, oxygen saturation meter, and built-in thermometer. It is envisaged that Epiwrist will also measure physical activity, sleep duration and patterns, blood pressure, and respiratory rate. The development of this software and hardware started in May 2020; it was designed by engineers at Softlution [30]. Although the development of Epiwrist is envisaged and a first prototype has been manufactured in China, it will require time and effort to perform real-life testing and obtain approvals.

During the COVID-19 epidemic, OHUs in hospitals were in charge of diagnosing health care workers with symptoms of the disease, and applying and changing protocols from their respective public health institutions, which included the study of contacts within the hospital, affecting both hospitalized patients and staff. OHUs also reported the cases to the local epidemiological surveillance systems. Moreover, OHUs participated in the constant updating and implementation of internal protocols for COVID-19 prevention in collaboration with the preventive medicine units. In our study, some hospitals showed an unwillingness to participate because they believed that HEpiTracker would interfere with established tracking of health care providers and surveillance. Moreover, they claimed that it could affect the privacy rights of participants. In general, public health interventions during infectious outbreaks can be divided into those consisting of personal actions (eg, physical distancing, personal hygiene, and use of protective equipment), case and contact identification (eg, test-trace-track-isolate, reactive school or workplace closure), regulatory actions (eg, governmental limits on sizes of gatherings or business capacity; stay-at-home orders; proactive school, workplace, and public transport closure or restriction; cordon sanitaire or internal border closures), and international border measures (eg, border closure or enforced quarantine).

### Conclusions

A key priority during the ongoing COVID-19 pandemic is to identify the combination of measures that minimizes societal and economic disruption while adequately controlling infection [31]. Our aim with HEpiTracker was therefore focused on case and contact identification, namely test-trace-track-isolate within hospital staff, as they were becoming infected with COVID-19 disproportionately more frequently and severely than the general population. The significance and impact of mobile apps, including HEpiTracker, in helping to tackle COVID-19 should be assessed further with more research conducted by other groups in real conditions. As we are facing a new virus and disease [32], future directions and scenarios should be further assessed [33].

HEpiTracker is an already available tool to monitor COVID-19 and other epidemics in hospital workers. It has been tested in real conditions and might represent a stepping stone toward effective health policies in response to future waves of the pandemic. HEpiTracker is available in Spanish, Portuguese, and English and holds the potential to become a customized asset to be used in future COVID-19 pandemic waves and other environments.

## Abbreviations

AMADIICH: Active Monitoring And Determinants of Incident Infection of COVID-19 in a Hospital population
CDC: Centers for Disease Control and Prevention
COPD: chronic obstructive pulmonary disease
HEpiTracker: Hospital Epidemics Tracker
ICO: Institut Català d'Oncologia
mHealth: mobile health
OHU: occupational health unit
PCR: polymerase chain reaction
VAS: visual analog scale
WHO: World Health Organization
